# Sleep Apnea Detection Based on Multi-Scale Residual Network

**DOI:** 10.3390/life12010119

**Published:** 2022-01-14

**Authors:** Hengyang Fang, Changhua Lu, Feng Hong, Weiwei Jiang, Tao Wang

**Affiliations:** 1 School of Computer and Information, Hefei University of Technology, Hefei 230009, China; 2019170907@mail.hfut.edu.cn (H.F.); lch6208@163.com (C.L.); 0120110560@czu.edu.cn (F.H.); 2 Intelligent Interconnected Systems Laboratory of Anhui Province, School of Computer and Information, Hefei University of Technology, Hefei 230009, China

**Keywords:** multi-scale, residual network, sleep apnea, ECG signals, focal loss

## Abstract

Aiming at the fact that traditional convolutional neural networks cannot effectively extract signal features in complex application scenarios, a sleep apnea (SA) detection method based on multi-scale residual networks is proposed. First, we analyze the physiological mechanism of SA, which uses the RR interval signals and R peak signals derived from the ECG signals as input. Then, a multi-scale residual network is used to extract the characteristics of the original signals in order to obtain sensitive characteristics from various angles. Because the residual structure is used in the model, the problem of model degradation can be avoided. Finally, a fully connected layer is introduced for SA detection. In order to overcome the impact of class imbalance, a focal loss function is introduced to replace the traditional cross-entropy loss function, which makes the model pay more attention to learning difficult samples in the training phase. Experimental results from the Apnea-ECG dataset show that the accuracy, sensitivity and specificity of the proposed multi-scale residual network are 86.0%, 84.1% and 87.1%, respectively. These results indicate that the proposed method not only achieves greater recognition accuracy than other methods, but it also effectively resolves the problem of low sensitivity caused by class imbalance.

## 1. Introduction

Sleep is necessary for everyone, and the quality of sleep directly affects people’s work and life. Humans spend lots of time sleeping, and sleep research has received a lot of attention because of the importance of quality sleep [1]. The American Academy of Sleep Medicine (AASM) divides sleep into five stages: wakefulness (W), N1, N2, N3 and REM. Among them, N1, N2 and N3 form the non-rapid eye movement (NREM) part of the sleep cycle, and the remaining stage is REM [1]. REM and NREM represent some important functions of the brain, including cell recovery, memory consolidation and brain metabolite clearance [2]. Sleep apnea (SA) is a common respiratory sleep disorder. Due to SA, the patient will experience symptoms such as decreased blood oxygen saturation and repeated awakenings during sleep, resulting in decreased sleep quality and even cardiovascular, metabolic abnormalities, neurocognitive disorders and other diseases [3,4,5,6]. According to the pathogenesis, SA can be divided into obstructive sleep apnea (OSA), central nervous sleep apnea (CSA) and mixed sleep apnea, of which OSA is the most common SA type.

In clinical practice, SA is usually detected by polysomnography (PSG), which is also the gold standard for SA diagnosis [7,8,9]. However, this method requires the patient to stay in a professional sleep laboratory for 1 to 2 nights. Sensors are used to collect electrocardiograph (ECG), respiratory signals and blood oxygen saturation (SpO_2_) and other physiological signals [10,11], and then SA is manually labeled. The detection process is complex and costly, making it impossible for many patients to be diagnosed and treated in a timely manner. Therefore, it has become a consensus of researchers to explore convenient and inexpensive methods of detecting SA.

After an extensive analysis of many physiological signals related to sleep apnea, researchers find that when a breath apnea event occurs, the RR interval in the ECG signal changes periodically. For this reason, they proposed using single-channel ECG signals combined with machine learning to quickly detect sleep apnea. There are currently two types of SA detection methods based on single-channel ECG signals: models based on traditional machine learning and models based on deep learning. There are many typical patterns based on traditional machine learning. Pinho et al. [12] uses heart rate variability (HRV) features and ECG-derived respiration (EDR) features, combined with artificial neural networks (ANN) and support vector machines (SVM), to achieve SA detection. Viswabhargav et al. [13] uses EDR and sparse residual entropy (SRE) features, combined with fuzzy K-means clustering and SVM to detect SA. Feng et al. [14] uses unsupervised learning to extract feature sets and uses time-dependent cost-sensitive (TDCS) to achieve SA detection. Although these methods have achieved some results, their performance is largely influenced by the characteristics of the manual design. Sharma et al. [15] proposes a method based on biorthogonal antisymmetric wavelet filter bank (BAWFB).

In recent years, deep learning models have been receiving growing attention. Li et al. [16] proposed an SA detection method based on sparse auto-encoder and hidden Markov model (HMM). This method first uses an unsupervised sparse autoencoder to learn features, and then SVM is used to classify ECG signals. Urtnasan et al. [17] uses a convolutional neural network (CNN) composed of six optimized convolutional layers to implement the SA detection model. Compared with the model based on traditional machine learning, the model based on deep learning avoids the dependence on human-crafted features, but there are still some shortcomings.

Existing models based on CNN usually use single convolution kernels for feature extraction. However, in complex application scenarios, it is difficult for traditional convolutional to efficiently provide salient features. Meanwhile, there is a class imbalance in the SA database, which leads to the low sensitivity of the model. In order to resolve the above problems, the paper proposes a method of SA detection based on a multi-scale residual network. First, we analyze the physiological mechanism of SA and extract the derived RR interval signals and R peak signals of the ECG signals as input. Then, feature extraction is performed on the derived signals by using a multi-scale residual network to obtain sensitive features from different perspectives. Finally, a fully connected layer is used to achieve SA detection. In addition, a class imbalance in the database is put into consideration, and a focal loss function is adopted to replace the traditional cross-entropy loss function, so that the model focuses more on the learning of difficult samples in the training phase to reduce the impact of class imbalance. By testing on the Apnea-ECG database [16,17,18], the proposed multi-scale residual network obtained an accuracy of 86.0%, a sensitivity of 84.1% and a specificity of 87.1%. Compared with the existing work, the method not only obtains a better classification accuracy but also effectively solves the problem of low sensitivity caused by class imbalance.

## 2. Materials and Methods

### 2.1. Flow Diagram of the Work

**Figure 1:**
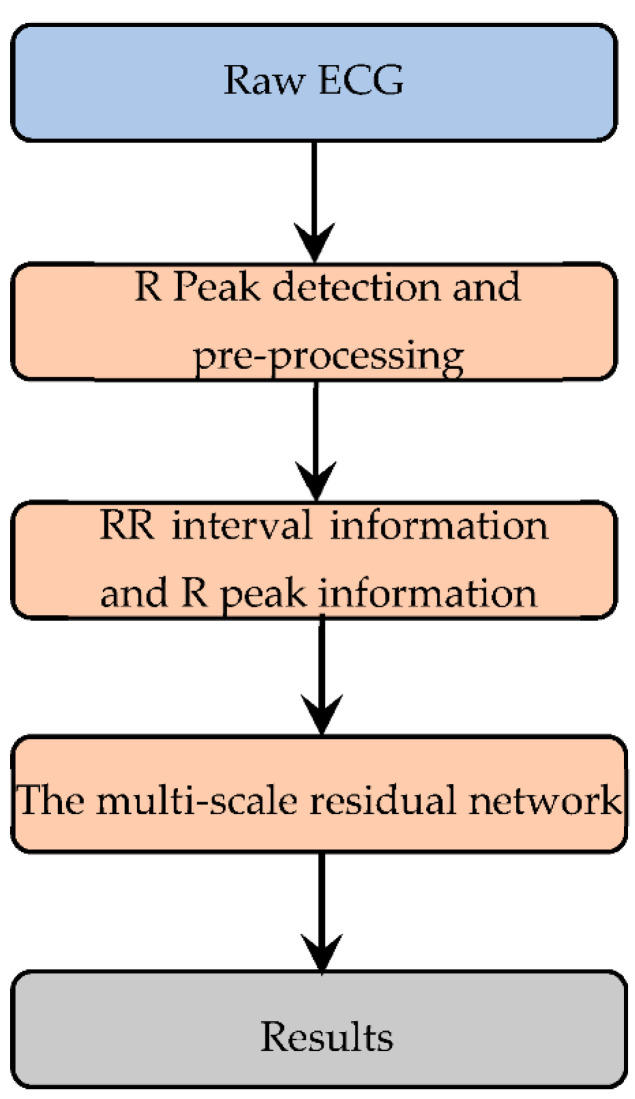
The process of the proposed method.

The flow diagram of the proposed method is shown in Figure 1. We obtained RR interval information and R peak information from the original signal through preprocessing, and then we used the proposed multi-scale residual network for feature extraction and classification [15,19].

### 2.2. Experimental Data

In this paper, the Apnea-ECG database [17,18] was used to verify the proposed method. The database has a total of 32 subjects, including 25 males and 7 females, and the age of the subjects is between 27 and 63 years old. The database consists of two data sets: a training set and a test set, with a total of 70 ECG signals records. The sampling rate is 100 Hz, and the sampling duration is between 401 and 578 min. The two data sets have a total of 34,313 min of signals, of which the training set contains 17,045 min, and the test set contains 17,268 min. After removing the abnormal ECG signals, segmentation was performed according to the 60 s segment, and finally 33,752 segments were retained, including 16,743 in the training set and 17,009 in the test set. According to the apnea-hypopnea index (AHI) value, 70 samples were divided into category A, category B and category C. When the sample’s AHI value was greater than 10, the sample was defined as type A. When the AHI value was greater than 5, the sample was defined as type B. When the AHI value was less than 5, it was defined as type C.

The UCD dataset is the second dataset used in this paper. It is collected by the University College Dublin. We used the UCD dataset to verify the generalization performance of the proposed method. The UCD dataset contains the overnight PSG of 25 patients, with subjects ranging in age from 28 to 68 years old [20].

### 2.3. Signal Denoising

The clinically collected ECG signals are very weak electrical signals, which are easily interfered with by the collection equipment and external noise. In order to ensure the accuracy of the SA detection method, it is necessary to filter out the relevant noise before further processing [16]. Common noises in ECG signals include the following:

- Baseline wandering—It is mainly caused by the low-frequency interference signals caused by poor contact of the measuring electrode or the patient’s breathing [21]. The frequency is between 0.05 Hz and 2 Hz, indicating that the ECG signals deviate from the normal baseline position.
- Power line interference—It is mainly 50 Hz/60 Hz noise generated by the power system, which will cause the entire waveform to be ambiguous and have a greater impact on the waveform.
- Electromyography noise—It is mainly caused by muscle fibrillation and contraction. The amplitude is small and the frequency is high [22]. The frequency is between 5 Hz and 2000 Hz, presenting an irregular and rapidly changing waveform.

Commonly used filtering methods include wave transformation, adaptive filtering, IIR filter, FIR filter, artificial neural network, etc. These methods have achieved good results in ECG signal analysis [18]. In this study, considering performance and speed, an FIR band pass filter of 3 Hz~45 Hz was selected to filter the ECG signals.

### 2.4. R Peak Location and Signal Extraction

**Figure 2:**
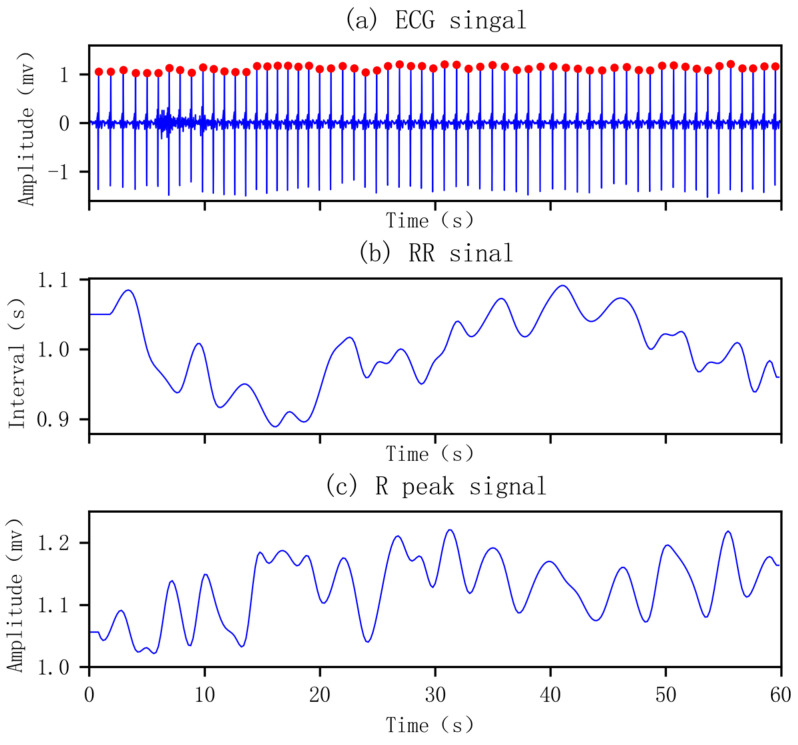
Positioned R peak and extracted derived RR interval signal and R peak signal.

Directly using ECG signals to detect SA could lead to model overfitting because it contains a lot of information which is unrelated to SA. This paper analyzes the physiological mechanism of SA and uses the derived signals of the ECG signals to extract features. However, before extracting the derived signals, the R peak position of the ECG signals should be determined first. Among the existing R peak positioning algorithms, the Pan–Tompkins algorithm proposed by Pan J and Tompkins W [23] has a high recognition rate and a good real-time performance, and it is widely used in clinical practice. Therefore, this paper uses the Pan–Tompkins algorithm (improved version of Hamilton et al.) [24] to determine the position of the R peak. Figure 2a shows the R peak identified by the Pan–Tompkins algorithm.

The rhythmic heartbeat reflects the balance between the sympathetic nervous system and the parasympathic nervous system [25,26]. When SA occurs, because of hypoxia, the concentration of carbon dioxide increases, and the sympathetic nerve will be activated, resulting in the breakdown of the balance between the sympathetic nervous system and the parasympathetic nervous system. Studies have shown that the RR interval of SA patients is longer than normal [9,27,28]. Therefore, compared with the direct use of ECG signals, the use of derived RR interval signals can more intuitively diagnose SA. The appearance time of the R peak is taken as the abscissa and the RR interval as the ordinate to draw the curve of RR interval and time, which is the RR interval signal.

In addition to the RR interval, the decrease in respiratory amplitude, the state of hypoxia and hypercapnia during SA will further strengthen the body’s respiratory movement, thus interrupting the regular fluctuations of the R peak [29]. Therefore, by analyzing the R peak of the ECG signals, SA can also be effectively diagnosed. Taking the appearance time of the R peak as the abscissa and the amplitude of the R peak as the ordinate, the curve of the R peak with time is drawn, which is the R wave signal. The obtained original RR interval signals and R peak signals are RR interval sequences with unequal time intervals, which need to be converted into equal time interval signals before further use. Referring to the existing research, this paper uses cubic spline interpolation to interpolate it, and the extracted RR interval signals and R wave signals are shown in Figure 2b,c.

### 2.5. Residual Network

**Figure 3:**
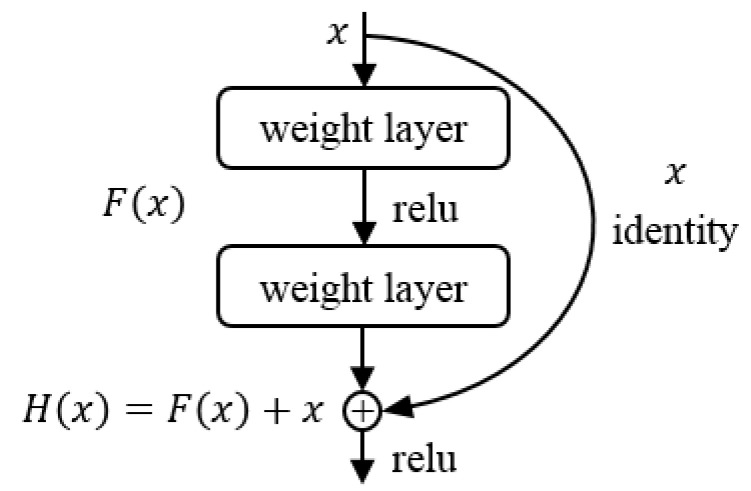
Residual block.

In theory, increasing the depth of the CNN will further enhance the expressive ability of the model. However, in actual research, researchers find that as the network deepens, network degradation may occur during the training process. He et al. [30] proposed the concept of residual network (ResNet). ResNet is composed of a stack of residual blocks. The feature extraction ability of the model is improved by adding “shortcut” connections to the residual blocks, thus solving the problem of network degradation caused by the deepening of the network. A typical residual block structure is shown in Figure 3. 

### 2.6. Construction of Multi-Scale Residual Network Model

**Figure 4:**
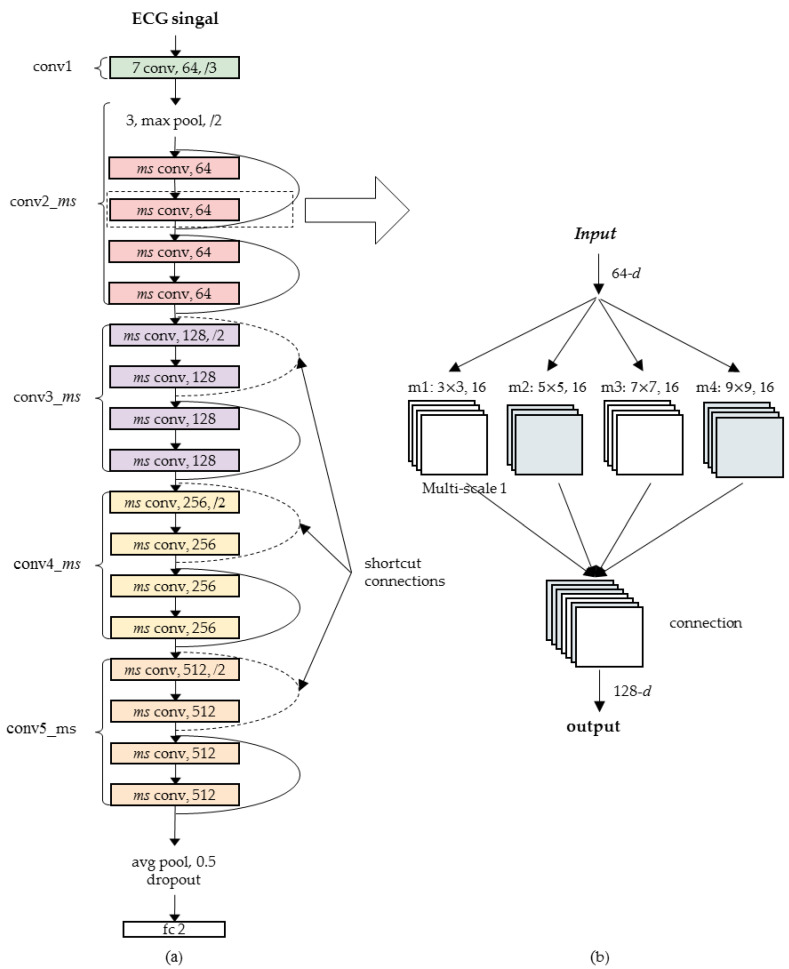
Multi-scale residual network. (**a**) Network structure; (**b**) multi-scale block.

Traditional residual networks usually use a single convolution kernel for feature extraction. However, in complex application scenarios, it will cause the model to omit local important features when adaptively selecting features, resulting in reduced accuracy of the model. Aiming at solving the shortcomings of traditional residual networks, this paper proposes a multi-scale residual network model. The multi-scale is mainly reflected in the network, which adopts convolution kernels of multiple scales to perform convolution operations simultaneously to extract sensitive features from different perspectives and improve the model prediction accuracy. Figure 4 shows the specific structure of the proposed multi-scale residual network.

The network we proposed is based on an 18-layer ResNet network model, which enhances the feature presentation ability of the network by adding multiple scales. Table 1 lists the detailed structural parameters of the network. The residual blocks corresponding to conv2_ms, conv3_ms, conv4_ms and conv5_ms are 2, 2, 2 and 2. Each residual block contains 2 layers, and each layer is composed of 4 convolution kernels of different scales. There are a total of 16 layers of residual units, which are 18 layers including conv1 and fully connected layers. It is worth mentioning that both the RR interval signals and the R peak signals are one-dimensional signals, so the multi-scale ResNet18 used in this paper is one-dimensional convolution. At the same time, in order to reduce the risk of network overfitting, a random dropout of 0.5 is set between the fully connected layer and the residual network.

### 2.7. Data Imbalance Processing

In SA detection, there is a significant difference in the number of abnormal ECG signal fragments and normal ECG signal fragments, which will lead to the tendency to learn simple counterexample samples (normal ECG signals fragments) during model training. In order to solve the above problems, a class weight is usually set in the cross-entropy loss function to balance the positive and negative examples. The formula is as follows:

(1) $$CE\left(p_{t}\right)=-\alpha_{t}\log\left(p_{t}\right)$$

where $\alpha_{t}$ represents the weight of the category, which is between 0 and 1. Although this method improves the tendency of the model in the training process to a certain extent, it does not solve the problem of the difficulty of classification in different samples caused by sample imbalance.

In order to solve this problem, Lin et al. [31] proposed a focal loss function based on the cross-entropy loss function. The formula is as follows:

(2) $$FL\left(p_{t}\right)=-\alpha_{t}{\left(1-p_{t}\right)}^{\gamma }\log\left(p_{t}\right)$$

Compared with the cross-entropy loss function, the focal loss function introduces a modulation factor ${\left(1-p_{t}\right)}^{\gamma }$, which reduces the weight of easy-to-separate samples, makes the model pay more attention on training difficult-to-separate samples, and improves the accuracy of classification. When γ=0, the loss function is the ordinary cross-entropy loss function with category weight, which only solves the tendency problem of the model. When γ>0, the modulation factor plays a role, and the network model focuses on misclassified samples. The larger the γ, the more attention is paid to the misclassified samples.

## 3. Experiment and Result Analysis

### 3.1. Sleep Apnea Detection Experiment

In this paper, five indicators which are accuracy, sensitivity, specificity, area under the curve (AUC) and F1-scroe were used to evaluate our proposed method.

This paper verifies the proposed method based on the Apnea-ECG database, where the training set is used to train the model, and the test set is used to evaluate the model. Table 2 lists the performance of this method on the test set. It can be seen that the method proposed in this paper correctly detected 9158 segments from 10,511 normal ECG signal segments and 5462 segments from 6498 sleep apnea ECG signal segments. The overall accuracy rate reached 86.0%, indicating a high accuracy rate of sleep apnea detection.

Aiming at the fact that the traditional CNN cannot effectively extract signal features in complex application scenarios, this paper proposes a sleep apnea detection method based on multi-scale residual networks.

In order to verify the advantages of this method, this section analyzes the performance before and after adopting the multi-scale convolution topology, as shown in Table 3. The accuracy of the traditional ResNet was 84.6%, the sensitivity was 82.2%, the specificity was 86.1%, the AUC was 0.918 and the F1-score was 80.3. After adopting the multi-scale convolution topology structure, its accuracy, sensitivity, specificity, AUC and F1-score increased to 86.0%., 84.1%, 87.1%, 0.931 and 82.1. The results show that in the SA detection research, the use of a multi-scale convolution topology structure can more effectively extract the features in the original signals.

In clinical practice, abnormal data or cases are often far less than normal data or cases, resulting in a certain imbalance of data. This also has a similar problem in SA detection research where the ECG signal fragments with sleep apnea are usually far fewer than the normal ECG signal fragments. If the data are not processed, the performance of the method will be biased towards the type with more data (normal data or cases), and the disease cannot be effectively diagnosed. Class weight (class weight) is a common method to deal with data imbalance, but this method does not solve the problem of different sample classification difficulty caused by sample imbalance.

**Figure 5:**
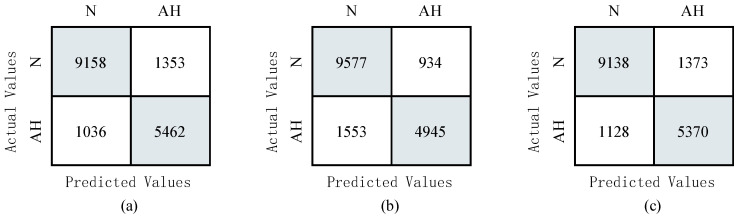
Performance comparison of focus loss function, class weight and without using any data imbalance technology. (**a**) The proposed method; (**b**) without using any data imbalance technology method; (**c**) class weight method.

This paper introduces a focus loss function to make the model focus on the learning of difficult samples in the training phase, thus reducing the impact of data imbalance. In order to verify the effectiveness of this method, we have compared it with the traditional method. It can be seen from Figure 5 that when no data imbalance technique was used, only 4945 ECG signal fragments were detected. Compared with the other two methods, the number of correctly detected sleep apnea ECG signal fragments was significantly reduced. Compared with the model using category weights, it can be found that the method using the focus loss function further improved the detection of sleep apnea ECG signal segments while maintaining the correct detection of normal ECG signal segments. This is because the focal loss function adjusts the loss of easy-to-classify samples, which forces the model to focus on the learning of difficult samples, reducing the impact of data imbalance.

### 3.2. Per-Recording Classification

According to the test results, we can further classify the tester and determine whether the tester has SA. According to the recommendations of the American Academy of Sleep Medicine (AASM), when the AHI value is greater than 5, the patient is considered to have SA. The definition of AHI is as follows:

(3) $$AHI=\frac{60}{T}*\mathrm{num}\;\mathrm{of}\;\mathrm{SA}\;\mathrm{segments}$$

T means the number of signals per minute, and L/60 is the number of hours for a recording. As shown in Table 4, the accuracy of the traditional ResNet was 91.2, the sensitivity was 100, and the specificity was 75. Compared with the traditional ResNet, the performance of the proposed multi-scale residual network was better, which had a 16% higher specificity. This paper also compares the AHI value predicted by the network with the actual AHI value in the database, and the results are shown in Table 4.

### 3.3. Test the Model on the UCD Database

In order to verify the generalization performance, the proposed model was evaluated on the UCD dataset. Since the UCD dataset has less data available for model learning, the performance of the proposed method on the UCD dataset was worse than that of the Apnea-ECG dataset. The experimental results are shown in Table 5, under the same preprocessing of the data set, and the performance of the proposed multi-scale residual network was better than that of the traditional ResNet. In general, the proposed method is useful for SA detection.

### 3.4. Comparison of Similar Research Results

In order to further verify the effectiveness of the method in this paper, we compared it with the same type of research work in recent years [10,12,13,14,16,32,33,34,35]. Table 6 lists the comparison results of this method and related work in terms of accuracy, sensitivity and specificity. It should be pointed out that in order to ensure the reliability of the results, the comparative work is evaluated based on the Apnea-ECG database. Due to the different pretreatment processes of different works, the claimed performance may be slightly different from the actual performance. It can be seen from Table 6 that the accuracy of existing machine learning-based methods is 78.1–85.1%, and the accuracy of detection methods based on deep learning is 2% higher. In addition, the method in this paper can obtain higher sensitivity while obtaining better specificity, while existing work usually sacrifices sensitivity or specificity to improve another index. In clinical practice, too low sensitivity or too low specificity is unacceptable, which will lead to a high rate of misdiagnosis. The method of multi-scale residual network combined with focus loss function proposed in this paper not only effectively improved the detection accuracy of sleep apnea, but it also effectively improved the problem of low sensitivity caused by data imbalance.

## 4. Conclusions

This paper proposes a sleep apnea detection method based on a multi-scale residual network. In this method, we use multi-scale convolution kernels to extract features at different levels, avoiding the limitations of the traditional single convolution topology. Considering that ECG signals contain a lot of information unrelated to sleep apnea, through the analysis of the physiological mechanism of sleep apnea, the derived RR interval signals and R peak signals in the ECG signals are extracted as the model input. In addition, in the study of sleep apnea detection, different types of ECG signal fragments have data imbalances. This study introduces a focus loss function to make the model focus more on the learning of difficult samples during the training phase to reduce the impact of data imbalance on performance. The experimental results on the public database Apnea-ECG show that the proposed method achieved an accuracy rate of 86.0%, a sensitivity of 84.1% and a specificity of 87.1%. Compared with existing work, the proposed method not only effectively improved the detection accuracy of sleep apnea, but it also effectively solved the problem of low sensitivity caused by data imbalance. Due to the limitations of the dataset used, the method proposed in this paper cannot distinguish between hypopnea and apnea. In the future, we will try to use wavelets to preprocess the proposed method, and we will verify it on other data sets many times to prove the generalization performance of the method.

## Figures and Tables

**Table 1:** Multi-scale residual network structure parameters.

Layer	Output Size	Network Architecture
conv1	100 × 1	Convolutional layer: 7 × 1, 64, Stride: 3
conv2_*ms*	50 × 1	Pooling layer: 3 × 1, Stride: 2
		${\left[\begin{array}{cc} {\left\{\begin{array}{c} 3\times 1,\;16\\ 5\times 1,\;16\\ 7\times 1,\;16\\ 9\times 1,\;16 \end{array}\right\}}_{C} & {\left\{\begin{array}{c} 3\times 1,\;16\\ 5\times 1,\;16\\ 7\times 1,\;16\\ 9\times 1,\;16 \end{array}\right\}}_{C} \end{array}\right]}_{T}\times 2$
conv3_*ms*	25 × 1	${\left[\begin{array}{cc} {\left\{\begin{array}{c} 3\times 1,\;32\\ 5\times 1,\;32\\ 7\times 1,\;32\\ 9\times 1,\;32 \end{array}\right\}}_{C} & {\left\{\begin{array}{c} 3\times 1,\;32\\ 5\times 1,\;32\\ 7\times 1,\;32\\ 9\times 1,\;32 \end{array}\right\}}_{C} \end{array}\right]}_{T}\times 2$
conv4_*ms*	13 × 1	${\left[\begin{array}{cc} {\left\{\begin{array}{c} 3\times 1,\;64\\ 5\times 1,\;64\\ 7\times 1,\;64\\ 9\times 1,\;64 \end{array}\right\}}_{C} & {\left\{\begin{array}{c} 3\times 1,\;64\\ 5\times 1,\;64\\ 7\times 1,\;64\\ 9\times 1,\;64 \end{array}\right\}}_{C} \end{array}\right]}_{T}\times 2$
conv5_*ms*	7 × 1	${\left[\begin{array}{cc} {\left\{\begin{array}{c} 3\times 1,\;128\\ 5\times 1,\;128\\ 7\times 1,\;128\\ 9\times 1,\;128 \end{array}\right\}}_{C} & {\left\{\begin{array}{c} 3\times 1,\;128\\ 5\times 1,\;128\\ 7\times 1,\;128\\ 9\times 1,\;128 \end{array}\right\}}_{C} \end{array}\right]}_{T}\times 2$
	1 × 1	Dropout: 0.5,
Computing power		0.144 × 10^9^

**Table 2:** The performance of the proposed method on the test set.

		Forecast Result			Accuracy/%	Sensitivity/%	Specificity/%
		N	AH	Total			
Realitylabel	N	9158	1353	10,511	86.0	84.1	87.1
	AH	1036	5462	6498			
	Total	10,194	6815	17,009			

**Table 3:** Performance comparison before and after using multi-scale convolution topology.

Method	Accuracy/%	Sensitivity/%	Specificity/%	AUC%	F1-Score/%
ResNet	84.6	82.2	86.1	0.918	80.3
ResNet + Multiscale	86.0	84.1	87.1	0.931	82.1

**Table 4:** Performance of ResNet + Multiscale and ResNet in per-recording classification.

Method	Accuracy/%	Sensitivity/%	Specificity/%	AUC	Corr/%
ResNet	91.2	100	75	0.985	0.945
ResNet + Multiscale	97.1	100	91.7	1	0.956

**Table 5:** The performance of our proposed model on the UCD dataset.

Method	Accuracy/%	Sensitivity/%	Specificity/%
ResNet	67.1	35.5	72.2
ResNet + Multiscale	72.4	36.5	83.6

**Table 6:** The performance comparison between the proposed method and similar research.

Work	Method	Accuracy/%	Sensitivity/%	Specificity/%
Sharma and Sharma	LS-SVM	83.4	79.5	88.4
Pinho et al.	ANN/SVM	82.1	88.4	72.3
Viswabhargav et al.	SVM	78.1	78.0	78.1
Surrel et al.	LS-SVM	82.2	73.3	87.6
Li et al.	DNN + HMM	84.7	88.9	82.1
Feng et al.	TDCS	85.1	86.2	84.4
Martin-Gonzalez et al.	LDA + QDA + LR	84.8	81.5	86.8
Chang et al.	1D CNN	87.9	81.1	92.0
Singh et al.	CNN + Decision Fusion	86.2	90.0	83.8
Our method	ResNet + Multiscale	86.0	84.1	87.1

## Data Availability

Data used in this work is open-source and publicly available on PhysioNet, https://physionet.org/content/apnea-ecg/1.0.0/ and https://physionet.org/content/ucddb/1.0.0/ (accessed on 10 January 2022).
